# Tumor‐associated N1 and N2 neutrophils predict prognosis in patients with resected pancreatic ductal adenocarcinoma: A preliminary study

**DOI:** 10.1002/mco2.183

**Published:** 2022-11-03

**Authors:** Hanlin Yin, Shanshan Gao, Qiangda Chen, Siyao Liu, Sami Shoucair, Yuan Ji, Wenhui Lou, Jun Yu, Wenchuan Wu, Ning Pu

**Affiliations:** ^1^ Department of General Surgery Zhongshan Hospital Fudan University Shanghai China; ^2^ Department of Radiology Zhongshan Hospital Fudan University Shanghai China; ^3^ Department of Surgery MedStar Health Baltimore Maryland USA; ^4^ Department of Pathology Zhongshan Hospital Fudan University Shanghai China; ^5^ Departments of Surgery, Medicine and Oncology Johns Hopkins University School of Medicine Baltimore Maryland USA

Dear Editor,

1

Pancreatic ductal adenocarcinoma (PDAC) has been widely considered as one of the most lethal malignancies with a 5‐year overall survival (OS) rate of only 11%.^1^ Lots of efforts have been devoted into its early diagnosis, treatment strategies, and pathogenesis, etc. PDAC is characterized as excessive desmoplastic tumor with abundant immune cells infiltration, mainly including macrophages, T cells, immature myeloid cells, and neutrophils. Neutrophils are the most abundant white blood cells in blood and have been regarded as a homogeneous group in the past, which belongs to innate immune system and participates in defensing against pathogens. Recently, accumulating evidences determined that neutrophils had an important role in PDAC and accounted for a substantial proportion of tumor‐infiltrating immune and inflammatory cells. Jiang et al.^2^ found that increased neutrophil infiltration was discovered as a central and prominent affected feature, which occurred in the liver, lung, and stomach at the PanIN stage. Importantly, serum leukotriene B4 (LTB4), derived from neutrophils, was validated for the early detection of PDAC. Considering its prognostic role, Ino et al.^3^ described relationships between prognosis and infiltrating immune cells in a cohort enrolled 212 PDAC patients who received radical surgical resection and found that tumor‐infiltrating neutrophils were positively correlated with macrophages and regulatory T cells infiltrations and closely associated with shorter OS and disease‐free survival (FS). This indicated that high tumor‐infiltrating neutrophils always present boost immunosuppressive microenvironment and contribute to poor survival. Apart from tumor‐infiltrating neutrophils, higher circulating neutrophils have also been found a negative association with outcomes. In our previous report, we noted that circulating neutrophil counts, whether 3 days within preoperation or 1 day after operation, were associated with shorter recurrence‐FS (RFS) but not with OS, which may hinted that neutrophils exerted a pro‐tumor effect in PDAC.^4^

Tumor‐infiltrating neutrophils were often referred as tumor‐associated neutrophils (TANs) in most studies. Wang et al.^5^ identified a pro‐tumor subcluster of neutrophils in PDAC and uncovered the pro‐tumor mechanisms of TANs in PDAC microenvironment and revealed the association between high glycolytic activity and pro‐tumor functions in TANs. Similar to M1/M2 nomenclature of macrophages, recent studies also suggested that TANs could be divided into N1 and N2 categories based on their distinctive phenotypes: antitumorigenic N1 neutrophils and pro‐tumorigenic N2 neutrophils. According to current in vivo/vitro studies, the N1/N2 neutrophil polarization may be depended on the special cytokine milieu, mainly including interferon‐β (IFN‐β) and TGF‐β. However, little is known about the prognostic value of tumor‐associated N1/N2 neutrophils in PDAC.

We preliminarily collected the PDAC tissues after radical surgery from January 2012 to December 2015 in our institute and stained for tumor‐associated N1 and N2 neutrophils as refered.^6^, ^7^ The typical cell markers of tumor‐associated N1 neutrophils were MPO^+^CD11b^+^CD206^−^, and those of tumor‐associated N2 neutrophils were MPO^+^CD11b^+^CD206^+^ by three‐color immunofluorescence (IF) staining (Figure 1A). Studies have reported that N1 neutrophils may possess powerful antitumor properties through antibody‐dependent or direct cytotoxicity^8^, ROS‐mediated coupling^9^, etc. On the contrary, N2 neutrophils contributed to tumor angiogenesis by secreting vascular endothelial growth factor and matrix metallopeptidase 9, etc. and suppressing CTLs function by arginase.^10^ In this study, a total of 77 PDAC patients were preliminarily enrolled. Their median age was 65.0 [interquartile range (IQR) 59.5–70.0] years old, and 63.6% of patients (49 of 77) were male. Approximately 25% and 40% of PDAC patients have reached advanced T stages (T3 and T4) and aggressive N stages (N1 and N2) when surgery, respectively. Other clinicopathological characteristics are displayed in Table S1.

**FIGURE 1:**
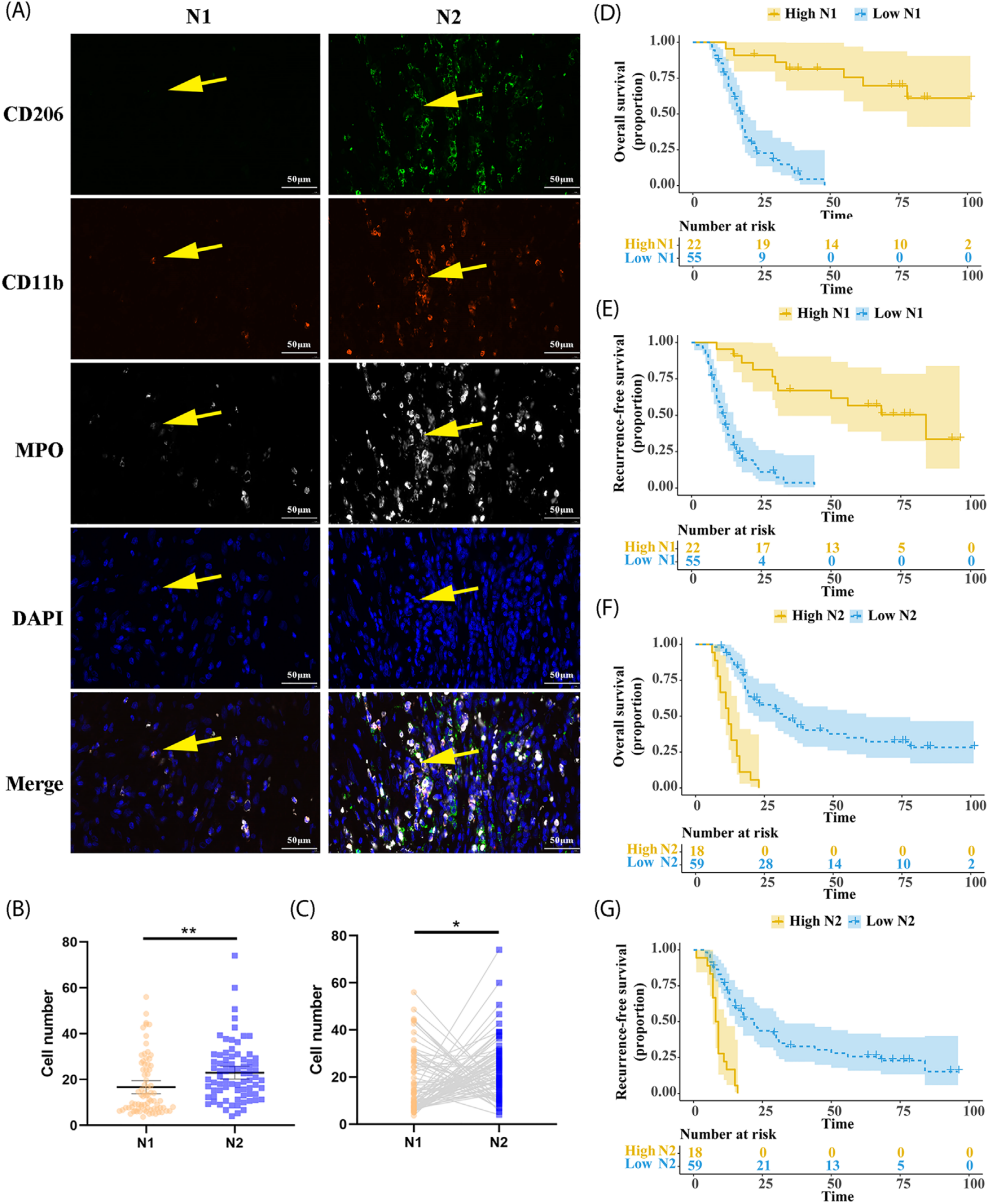
Identification and prognosis of the tumor‐associated N1 and N2 neutrophils within pancreatic ductal adenocarcinoma (PDAC). (A) Representative images of IF staining for tumor‐associated N1/N2 neutrophils. The left row displayed typical CD11b^+^MPO^+^CD206^−^ cells, which indicated N1‐polarized tumor‐associated neutrophils (TANs). The right row showed CD11b^+^MPO^+^CD206^+^ cells, which indicated N2‐polarized TANs. (B) A number comparison of tumor‐associated N1 and N2 neutrophils by unpaired analysis. (C) A number comparison of tumor‐associated N1 and N2 neutrophils by paired analysis. (D and E) The Kaplan–Meier survival depicting overall survival (OS) and recurrence‐free survival (RFS) according to the abundance of tumor‐associated N1 neutrophils. (F and G) The Kaplan–Meier survival depicting OS and RFS according to the abundance of tumor‐associated N2 neutrophils

The median number of tumor‐associated N1 and N2 neutrophils were 10.7 (IQR 7.2–25.2) and 21.0 (IQR 13.7–29.0), respectively. The unpaired analysis indicated that the mean number of tumor‐associated N2 neutrophils was 23.0 higher than that of tumor‐associated N1 neutrophils (16.7, *p* = 0.002, Figure 1B). Additionally, the discrepancy between the tumor‐associated N1 and N2 neutrophils were also observed in the paired analysis (*p* = 0.011, Figure 1C). According to the optimal cutoffs of tumor‐associated N1 and N2 neutrophils, 22 and 18 cases were respectively classified into the high N1 and N2 neutrophil infiltration group. As shown in Table S1, the lower tumor‐associated N1 neutrophil infiltration was significantly associated with easier lymph node metastasis (*p* = 0.012) and higher TNM stage (*p* = 0.024), but not with other factors. The higher tumor‐associated N2 neutrophil infiltration was significantly correlated with distal location (*p* = 0.039) and easier lymph node metastasis (*p* = 0.039), similar to the previous study that more aggressive PDAC preferentially recruited more neutrophils.

The median OS and RFS of this cohort were 20 and 15 months, respectively. There were 81.6%, 34.1%, and 26.5% OS rates at 1, 3, and 5 years, respectively, while the 1‐, 3‐, and 5‐year RFS rates were 57.9%, 24.9%, and 19.6%, respectively. The Kaplan–Meier survival analyses showed a significant difference in median OS and RFS between the high tumor‐associated N1 neutrophil group and the lower group [OS: not reached versus 18 months, Log‐rank *p* < 0.001, HR = 0.153, 95% confidence interval (CI) 0.089–0.262, Figure 1D; RFS: 84 versus 12 months, Log‐rank *p* < 0.001, HR = 0.209, 95% CI 0.125–0.348, Figure 1E]. Accordingly, the median OS (12 versus 32 months, Log‐rank *p* < 0.001, HR = 5.352, 95% CI 2.066–13.870, Figure 1F) and RFS (8.5 versus 22 months, Log‐rank *p* < 0.001, HR = 3.852, 95% CI 1.660–8.941, Figure 1G) of the more markable tumor‐associated N2 neutrophil infiltration group were both statistically shorter. Meanwhile, the univariate and multivariate Cox analyses together with conventional clinicopathological variables demonstrated that both tumor‐associated N1 and N2 neutrophils were independent prognostic factors for OS (Table S2) and RFS (Table S3) with the opposite HR values.

Accumulative evidences showed that neutrophils had complex interaction with tumor microenvironment and played an important role in PDAC progression. The predictive values of neutrophil relevant biomarkers have been found in PDAC patients and regarded as potential therapeutic target. High density of total TANs infiltration always indicated poor outcome in PDAC, and current strategies of anti‐TANs mainly focused on depleting TANs entirely or blocking chemokines that functioned as TANs recruitment. Series of preclinical studies used Ly6G or CXCR2 to block TANs and had encouraging outcomes in different mouse models. However, these therapeutic strategies are fraught with difficulties for further clinical trials because of the vital function in defense pathogens of neutrophils.

It is worth to consider that TANs have the opposite phenotype between N1 and N2. Our study successfully found that both tumor‐associated N1 and N2 neutrophils displayed significant and opposite prognostic values within PDAC. The N1/N2 polarization may depend on different stimuli, including TGF‐β induces N2 phenotype whereas IFN‐β signaling polarizes neutrophils to N1 phenotype, and the causes of opposite effect in N1 and N2 neutrophil may include the secretions of different cytokines that orchestrate immune cells recruitment, different capability of arginase and proteases synthesis, and direct or indirect cytotoxicity, etc. The aforementioned results hinted that precisely eliminating N2 by specific markers or modified/polarized TANs into N1 would be a more efficacious strategy with less toxicity.

In summary, the exploration of neutrophil polarization and its correlation to clinical features in PDAC would significantly enhance our understanding of its pathophysiology and enable us to develop better treatment options.

## AUTHOR CONTRIBUTIONS

*Study conception and design*: NP and HY. *Acquisition of data*: HY, SG, QC, SL, and NP. *Analysis and interpretation*: HY, SG, QC, SL, SS, YJ, WL, JY, WW, and NP. *Drafting of manuscript*: NP and HY. *Critical revision*: HY, SG, QC, SL, SS, YJ, WL, JY, WW, and NP. *Statistical analysis*: HY, SG, QC, and NP. *Study supervision*: NP and WW. *Read and approved the final manuscript*: HY, SG, QC, SL, SS, YJ, WL, JY, WW, and NP.

## CONFLICT OF INTEREST

The authors declare no conflict of interest.

## ETHICS STATEMENT

The study protocol was reviewed and approved by the Ethics Committee of Zhongshan Hospital, Fudan University.

## Supporting information

Supporting information

## Data Availability

Data included in this study are available upon request by contact with the corresponding author.
