# Supplementary material for: Tumor‐associated N1 and N2 neutrophils predict prognosis in patients with resected pancreatic ductal adenocarcinoma: A preliminary study
Source: MedComm (2020). 2022 Nov 3;3(4):e183. doi: 10.1002/mco2.183 (PMC9632487; doi:10.1002/mco2.183)
Supplement: Supplementary file 1 — Supporting information [file MCO2-3-e183-s001.docx]

**Supplementary materials**

**Tumor-associated N1 and N2 Neutrophils** **Predicts Prognosis in Patients with Resected Pancreatic Ductal Adenocarcinoma: A Preliminary Study**

Hanlin Yin, MD^1,2,#^, Shanshan Gao, MD, PhD^3,#^, Qiangda Chen, MD^1,2,#^, Siyao Liu^1,2,#^, Sami Shoucair, MD, MPH^5,8^, Yuan Ji, MD, PhD^2,4^, Wenhui Lou, MD, PhD^1,2^, Jun Yu, MD, PhD^5,6,7^, Wenchuan Wu, MD, PhD^1,2,*^, Ning Pu, MD, PhD^1,2,*^

*Department of ^1^General Surgery, ^3^Radiology and ^4^Pathology, Zhongshan Hospital, Fudan University, Shanghai, 200032, China*

*^2^ Cancer Center, Zhongshan Hospital, Fudan University, Shanghai, 200032, China*

*Departments of ^5^Surgery, ^6^Medicine and ^7^Oncology, Johns Hopkins University School of Medicine, Baltimore, MD, 21287, USA*

*^8^ Department of Surgery, MedStar Health, Baltimore, MD, 21218, USA*

^#^ These authors contributed equally to this work.

**Materials and Methods**

**Patient selection and clinical data**

All patients in our study were performed with pancreaticoduodenectomy (PD) or distal pancreatectomy (DP) at our hospital from January 2012 to December 2015, and pathologically diagnosed as PDAC. All formalin fixed paraffin-embedded (FFPE) tissues were preserved and collected from Department of Pathology. The inclusion and exclusion criteria as follows: i. All patients with complete clinical data, ii. no history of other malignant cancers, iii. no distant metastases or undetermined origins and receiving R0 resection, iv. without neoadjuvant therapies and adjuvant immune or targeted therapies, etc.

Clinical variables were collected containing gender, age, primary site, tumor differentiation, serum tumor markers, albumin, AJCC T stage, AJCC N stage, microvascular invasion, and follow-up data.

**Immunofluorescence (IF) staining**

On the FFPE tissues, we performed IF staining with anti-CD206 (Abcam), anti-CD11b (Abcam), and anti-MPO (Abcam, Cambridge, UK) monoclonal antibodies as the primary antibodies. Then, the Alexa Fluor^®^ 488, Alexa Fluor^®^ 647, Alexa Fluor^®^ 555 Tyramide Reagents (ThermoFisher Scientific, Waltham, DE, USA) and DAPI (Solarbio) were utilized as previously reported.

**Statistical analysis**

The statistical analyses in this study were operated by SPSS 21.0 (SPSS Inc., Chicago, IL, USA). The optimal cut-off values for tumor-associated N1 and N2 neutrophils were determined by the receiver operating characteristic (ROC) curves. The correlations between clinical variables and N1/N2 neutrophils were analyzed by the Pearson chi-square test or Fisher’s exact test, and the continuous variables presented as medians with interquartile range (IQR) were compared using the Student’s t-test and Paired-samples t-test. The overall survival (OS) was calculated from the date of surgery to death for any reason or was censored at the last follow-up. The recurrence-free survival (RFS) was calculated from the date of surgery to recurrence of disease, death, or was censored at the last follow-up. The log-rank test was utilized for Kaplan–Meier curves, and the Cox proportional hazards regression analysis was used in the univariate and multivariate analyses. Statistical significance was accepted with a p value <0.05 in a 2-tailed analysis.

**Supplementary Table 1. Relationships between tumor-associated N1 and N2 neutrophils and clinicopathological characteristics.**

| **Variables** | **Low N1** | **High N1** | ***P* value** | **Low N2** | **High N2** | ***P* value** |
| --- | --- | --- | --- | --- | --- | --- |
|  | **(n=55)** | **(n=22)** |  | **(n=59)** | **(n=18)** |  |
| **Gender** |  |  | 1.000 |  |  | 0.387 |
| Male/ Female | 35/20 | 14/8 |  | 36/23 | 13/5 |  |
| **Age (years)** |  |  | 0.149 |  |  | 0.950 |
| <65/ ≥ 65 | 30/25 | 8/14 |  | 29/30 | 9/9 |  |
| **Primary site** |  |  | 0.311 |  |  | **0.039** |
| Head/ Body or tail | 32/23 | 10/12 |  | 36/23 | 6/12 |  |
| **Differentiation** |  |  | 0.092 |  |  | 0.533 |
| I/ II/ III | 1/14/40 | 0/11/11 |  | 1/21/37 | 0/4/14 |  |
| **Microvascular invasion**  No/Yes | 50/5 | 21/1 | 0.840 | 54/5 | 17/1 | 1.000 |
| **T stage** |  |  | 0.155 |  |  | 0.199 |
| T1+T2/ T3+T4 | 39/16 | 19/3 |  | 47/12 | 11/7 |  |
| **N stage** |  |  | **0.012** |  |  | **0.039** |
| N0/N1-2 | 28/27 | 18/4 |  | 39/20 | 7/11 |  |
| **TNM stage**  I/ II/ III | 21/24/10 | 16/5/1 | **0.024** | 32/19/8 | 5/10/3 | 0.136 |
| **CA19-9** |  |  | 0.318 |  |  | 0.758 |
| < 37/≥37 U/L | 10/45 | 7/15 |  | 14/45 | 3/15 |  |
| **CEA** |  |  | 0.828 |  |  | 0.339 |
| < 5/ ≥ 5 ng/mL | 42/13 | 18/4 |  | 44/15 | 16/2 |  |
| **Albumin** |  |  | 0.505 |  |  | 0.410 |
| < 35/ ≥ 35 g/L | 13/42 | 3/19 |  | 14/45 | 2/16 |  |
| **Tumor-associated N1 neutrophils**  Low/ High | / | / | / | 38/21 | 17/1 | **0.014** |
| **Tumor-associated N2 neutrophils**  Low/ High | 38/17 | 21/1 | **0.014** | / | / | / |

**Supplementary Table 2. Univariate and multivariate analysis of prognostic factors for overall survival.**

| **Variables** | **Overall survival** | | |
| --- | --- | --- | --- |
|  | **Univariate *P* value** | **Multivariate *P* value** | **Multivariate HR (95% CI)** |
| **Gender** |  |  |  |
| Male/Female | 0.802 | NA |  |
| **Age (years)** |  |  |  |
| <65/≥65 | 0.146 | NA |  |
| **Primary site** |  |  |  |
| Head/Body or tail | 0.676 | NA |  |
| **Differentiation** |  |  |  |
| I/II/III | 0.071 | NA |  |
| **T stage** |  |  |  |
| T1+T2/ T3+T4 | 0.071 | NA |  |
| **N stage** |  |  |  |
| N0/N1-2 | **<0.001** | 0.121 | 1.838 (0.852-3.963) |
| **TNM stage** |  |  |  |
| I/II/III | **<0.001** | 0.828 | 0.944 (0.563-1.583) |
| **Microvascular invasion**  No/Yes | 0.167 | NA |  |
| **CA19-9** |  |  |  |
| < 37/ ≥ 37 U/L | 0.747 | NA |  |
| **CEA** |  |  |  |
| < 5/ ≥ 5 ng/mL | 0.769 | NA |  |
| **Albumin** |  |  |  |
| < 35/ ≥ 35 g/L | 0.362 | NA |  |
| **Tumor-associated N1 neutrophils** |  |  |  |
| Low/ High | **<0.001** | **<0.001** | 0.094 (0.030-0.293) |
| **Tumor-associated N2 neutrophils** |  |  |  |
| Low/ High | **<0.001** | **<0.001** | 3.973 (2.049-7.704) |

**Supplementary Table 3. Univariate and multivariate analysis of prognostic factors for recurrence-free survival.**

| **Variables** | **Recurrence-free survival** | | |
| --- | --- | --- | --- |
|  | **Univariate *P* value** | **Multivariate *P* value** | **Multivariate HR (95% CI)** |
| **Gender** |  |  |  |
| Male/Female | 0.628 |  |  |
| **Age (years)** |  |  |  |
| <65/≥65 | 0.298 |  |  |
| **Primary site** |  |  |  |
| Head/Body or tail | 0.871 |  |  |
| **Differentiation** |  |  |  |
| I/II/III | 0.118 |  |  |
| **T stage** |  |  |  |
| T1+T2/ T3+T4 | 0.242 |  |  |
| **N stage** |  |  |  |
| N0/N1-2 | **<0.001** | 0.060 | 2.048 (0.970-4.321) |
| **TNM stage** |  |  |  |
| I/II/III | **0.002** | 0.801 | 0.938 (0.570-1.543) |
| **Microvascular invasion**  No/Yes | 0.193 |  |  |
| **CA19-9** |  |  |  |
| < 37/ ≥ 37 U/L | 0.685 |  |  |
| **CEA** |  |  |  |
| < 5/ ≥ 5 ng/mL | 0.879 |  |  |
| **Albumin** |  |  |  |
| < 35/ ≥ 35 g/L | 0.329 |  |  |
| **Tumor-associated N1 neutrophils** |  |  |  |
| Low/ High | **<0.001** | **<0.001** | 0.136 (0.055-0.334) |
| **Tumor-associated N2 neutrophils** |  |  |  |
| Low/ High | **<0.001** | **0.004** | 2.583 (1.347-4.955) |
